# Methods of Wood Volume Determining and Its Implications for Forest Transport

**DOI:** 10.3390/s22166028

**Published:** 2022-08-12

**Authors:** Tadeusz Moskalik, Łukasz Tymendorf, Jan van der Saar, Grzegorz Trzciński

**Affiliations:** 1 Department of Forest Utilization, Institute of Forest Sciences, Warsaw University of Life Sciences–SGGW, 159 Nowoursynowska St., 02-776 Warsaw, Poland; 2 Faculty of Forestry, Warsaw University of Life Sciences–SGGW, 159 Nowoursynowska St., 02-776 Warsaw, Poland

**Keywords:** gross vehicle weight (GVW), wood volume determination, photo-optical measurements, machine vision, round wood weight, weight of wood load

## Abstract

Proper measurements are extremely significant for the forest owner, the harvesting company, the hauler, the final buyer, and the wood processing company. The accuracy of round wood volume determination is of fundamental importance in planning and accounting for individual processes related to the wood trade. It is the basis for determining the maximum quantity in single load of wood that allows for using the permissible total gross vehicle weight. The determination of wood load in cubic meters does not allow unequivocally determining its weight, which often leads to overloading of vehicles. This paper presents a comparison of the photo-optical method for determining the volume of wood to be transported with the real measurement and determination of the weight of a load and the total gross vehicle weight (GVW) with the simultaneous application of conversion factors determining the weight of the load from the volume of wood. The measurement included 23 broadleaf round wood piles (193.73 m^3^) and 14 coniferous round wood piles (149.23 m^3^). The measurement error for broadleaf wood piles ranges from −47.67% to 63.16%, and from −43.31% to 24.72% for coniferous wood piles. Determination of the volume of a broadleaf wood pile using the iFOVEA method had an average error of 1.34%, while the Timbeter method had an average error of −1.83%. In the coniferous wood pile measurement, the error is −12.82% and 2.41%, respectively. Verification of the volume of the large-sized wood indicated on the delivery note (reference value) on the log sorting line (by laser scanning) showed larger volumes by 0.10 m^3^ to 2.54 m^3^, giving a percentage error of 0.35% and 8.62%, respectively. As a consequence of the application of such methods for determining the weight of wood loads, the transport truck sets are often significantly overloaded, which has a significant impact on the accelerated degradation of roads and safety in traffic and timber transportation.

## 1. Introduction

Innovation activities with the development of digital techniques are increasingly affecting many areas of human activity, including forest management. An absolutely essential challenge for forest administration is the measurement and recording of harvested round wood. Wood measurement is an important process in the wood supply chain, which requires advanced solutions to cope with the current challenges [1]. The accuracy of determining the volume of round wood is of fundamental importance in planning and accounting for the individual processes involved in the wood raw material trade. At the same time, it is the basis for determining the maximum single load of wood for the total gross vehicle weight (GVW) of the transport tuck set [2,3,4].

The basis for the timber quality and dimensional classification are the standards in force in Poland, and the technical conditions issued by directives of the General Directorate of State Forests. They categorize particular assortments according to classification by quality and dimension [5,6]. The total volume of the removed wood consists of merchantable round wood, slash, and stump wood. The merchantable timber includes large-sized and medium-sized round wood.

Large-sized wood (L) is wood with a thin end diameter of 14 cm (excluding bark), calculated in single pieces. It is comparable to the assortment defined as sawmill wood. 

Medium-sized wood (S) is wood with a minimum diameter of 5 cm and more (excluding bark), with a thick end diameter of up to 24 cm, calculated in single pieces, in pieces as groups, and in stakes. In terms of quality and size, medium-sized wood is divided into four groups:

1. S1—timber wood; this group includes assortments classified according to its purpose as pitwood and wood for building props,
2. S2—stake wood for industrial processing (inter alia, pulpwood),
3. S3—round wood for industrial processing (perches), and
4. S4—firewood.

Determination of the volume of large-sized wood and medium-sized wood under Polish conditions has so far been carried out in a traditional (manual) manner. In the first case, a diameter gauge and a measuring tape are used. In the second case (for stack wood), the length, height and width of the stack must be determined with a measuring tape and the relevant calculations made. The necessary measurements take a lot of time [7].

In recent years, there has been an extremely rapid development of computer technology and mobile phones equipped with high-tech digital cameras.

For large-sized wood, further processing at the sawmills, general information on geometrical parameters of a given log (its length, upper, lower and middle diameter, shape, and volume) can be obtained from non-contact optical measuring techniques [8]. For wood log measurement, laser scanners are used for the most part, providing satisfactory accuracy, as well as scanning speed [9]. Such systems are those using two orthogonal scanners and measuring two orthogonal diameters in one cross-section of the log. In order to regain information on the entire dimension of the log, at least four laser scanners are required.

On the other hand, it is possible to measure woodpiles using photo-optical methods [10]. Recognition of stack dimensions from photographs is possible by knowing the resolution of the camera, the dimensions of a single pixel and the dimensions of the reference point. The measurement technology is based on the analysis of the captured stack images using appropriate algorithms [11,12,13,14]. The functioning of the different applications is quite similar and proceeds in the following phases:

1. Taking a series of images of a stack of medium or large-sized of round wood.
2. Merging of the images into one panoramic image.
3. Analysis of the panoramic image in order to:
  - Identification of the cross-cut forehead of round wood and counting them,
  - Inscribe circles in the cross-cut forehead sections,
  - Make a diameter distribution table,
  - Inscribing the pile into the polygon and calculating the area of the polygon, and
  - Determination of the replacement coefficient from the ratio of the area of the cross-cut foreheads of wood to the area of the polygon or use of a preset coefficient.
4. Calculation of the stack volume and preparation of the report.

The calculation of the volume of roundwood can be based on a designated replacement factor or a table factor determined for the country, species and length of the wood assortments. Currently, several applications are available on the European market, for example: (1) AFoRS, (2) Fovea, (3) Logsize, (4) LogStackPro, (5) mScale, (6) sScale, (7) Timbeter, and (8) Trestima Stack. Their accuracy, when measured correctly, generally does not exceed ±2%, compared to manual measurement according to current standards [15,16,17,18].

The transported round wood is characterized by a high variability of the different assortments, made of different species, and by the variable moisture content of the wood, which does not allow its weight to be clearly determined [19,20,21]. The impossibility of determining the precise weight of a single load of round wood is a reason for overloading of the transport sets beyond the legal limits [3,22,23,24,25]. In the research articles on factors influencing the weights of transported round wood as well as gross vehicle weight (GVW), the authors mainly mention wood assortment and tree species [17,21,26,27] as well as season of the year [3,24,25,26]. The moisture content of wood, which influences the density of 1 m^3^ of the load, is extremely important in this case and depends on the season of the year as well as on the part of the trunk from which the wood assortment comes [28,29,30,31].

An important aspect that affects the maximum volume of transported round wood is the legal provisions defining the permissible total gross vehicle weight of the transport set [4,32,33,34,35]. In Poland, there are regulations limiting the gross vehicle weight (GVW) of road transport set vehicle combinations, which depends on the number of axles and their propulsion. For five- or six-axle transport vehicle combinations, it is 40 Mg [36]. In some EU countries, GVW has been increased to 60 Mg or 76 Mg or even 92 Mg [25,37,38,39,40], and currently Finland and Sweden are piloting even longer and heavier vehicles, with a maximum weight of up to 104 Mg and a maximum length of up to 34.5 m [41].

Due to the problems of determining the real weight of a load of round wood, during loading onto transport truck set, legislation has been introduced in Poland and defining GVW as the weight of an empty transport set and the weight of the round wood load calculated by multiplying volume of wood in cubic meters and the normative density factor [42,43,44]. This means that, with the known weight of an empty truck transport set, the driver can load, and the forester can dispatch, such a volume of round wood that, after converting cubic meters to the weight of the load in kilograms, the GVW does not exceed the 40 Mg implied by the law.

Authors of studies of round wood truck transport in Poland point out shortcomings and discrepancies in GVW determined by law and the real weight obtained from weighing transport sets on stationary scales [25,45,46]. They point out, that one of the factors affecting the GVW is an error most probably due to the way the load volume is determined according to the PN-93/D-02002 [47,48] and Ordinance No. 51 z 2019 [5].

Specifying a load of round wood in cubic meters does not make it possible to clearly determine its weight, which often results in vehicles being overloaded.

The aim of this study is to compare the determination of the volume of transported wood by photo-optical methods (for medium-sized wood—S2) and by laser scanning on the sawmill log sorting line (for large-sized wood—Ls) with the volume obtained from manual measurements, commonly used in the State Forests National Forest Holding in Poland, while analyzing the impact of these methods on the determination of the single round wood load and gross vehicle weight.

## 2. Materials and Methods

### 2.1. Round Wood Volume Measurement

The traditional measurement (manual) commonly used in Poland was used as a reference measurement for determining the volume of both large- and medium-sized wood. The measurement of large-sized wood is usually carried out individually. The measurement elements are: length (*l*) and central diameter (*d*) or upper diameter (dg). The length is measured to an accuracy of 5 cm, in the case of logs to 1 cm. The volume of the wood measured in individual pieces (*V*) must be determined to two decimal places using the central diameter of the piece in cubic meters (m^3^) based on the formula [49]:

(1) $$V=\frac{\pi \cdot d^{2}}{40000}\cdot l$$

where

*d*—central diameter without bark, in centimeters (cm); 

*l*—length of the log, in meters (m); 

*π*—3.14.

The elements for measuring wood calculated in stacks are length (l), stack height (h) and width (s). Measurements are taken with a tape or other measuring instrument. The length of the stack is considered to be the nominal length of the stacked wood. The length is determined to the nearest 1 cm. The width of the stack shall also be measured to the nearest 1 cm with a tape measure, determining this parameter between the last wood pieces (logs).

The stack height is to be measured perpendicularly from the bottom edge of the stack to the top edge of the stack to the nearest 1 cm. The height of the stack, for each side, shall be determined as the arithmetic mean of at least four measurements. It is permissible to measure the height from one side of the stack. The measuring points should be evenly distributed along the width of the stack and permanently marked. The distances between measurements should not be greater than 1 m (for piles up to 10 m wide) and 2 m (for piles wider than 10 m). The first height measurement of a stack starts with no less than two stacked logs. A similar rule applies to the last measurement.

The volume of stacked wood must be determined to two decimal places. Stacked wood volume (*V*) is calculated in cubic meters (m^3^). To convert spatial cubic meters (m3p) into solid cubic meters (m^3^), the conversion factor x is needed. In Poland, different factors depending on three species and length are implemented by State Forest Service [50]. The final calculation is based on the formula:

(2) $$V=V_{p}\cdot x$$

(3) $$V_{p}=l\cdot s\cdot h$$

where 

*Vp*—stack volume in spatial cubic meters (m^3^p), 

*x*—the conversion factor according to the coefficients in the appropriate technical round wood conditions, 

*l*—length of the stack (m),

*s*—width of the stack (m), and

*h*—height of the stack (m).

Two applications, were used to determine the stacked wood volume, using photo-optical methods iFOVEA [51] and Timbeter [52].

**Figure 1:**
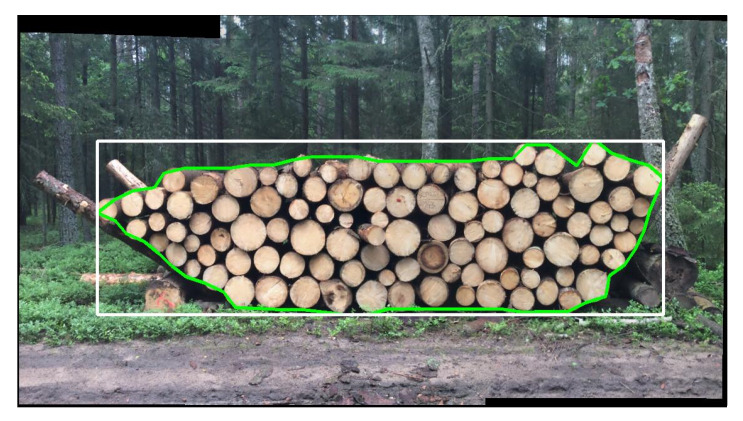
Identification of a stacked wood polygon by the iFovea application. (Fot. W. Sarzyński).

First, iFOVEA (SDP Digitale Produkte GmbH, Waiblingen, Germany), allows measurements to be taken on Android and iOS smartphones. To take a measurement, a photo of the stack must be taken and, in the case of a particularly large pile, the application offers the possibility of creating a panorama consisting of up to 35 photos. The reference value in this case is the width of the stack, which must be measured with a tape measure. The application can buffer the data offline and send the measurement results to a server after reconnecting to the internet. It uses the stack images (Figure 1) to calculate spatial meters, cubic meters, log diameters and many additional wood stack data immediately and directly on the mobile device.

During timber measurement, the position (location) is automatically recognized and recorded by GPS. This makes it possible to visualize the map with the measured pile data on a smartphone.

**Figure 2:**
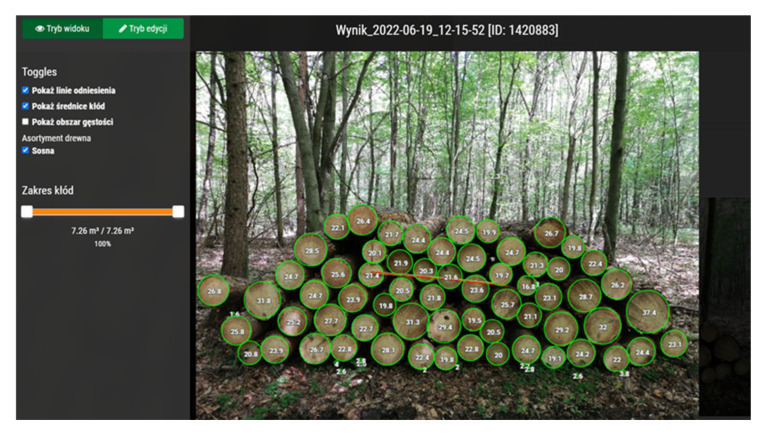
Automatic identification and determination of diameters of the stacked wood pieces by the Timbeter application. (Fot. T. Moskalik).

**Figure 3:**
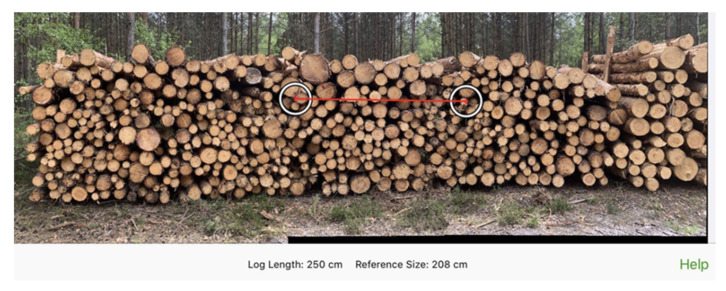
Setting the reference measurement in the Timbeter application. (Fot. M. Żak).

The Timbeter application (TIMBETER HQ, Tallinn, Estonia), as in the previous case, provides the possibility to determine the volume of a pile by analyzing a single image taken (Figure 2) or a series of images combined into a panorama. The reference value is an appropriately applied measure of known length (Figure 3 red line). The data obtained during the measurement are stored in an application on the device and, when connected to the internet, in the Timbeter memory module.

For 37 stacks, S2 timber volumes were measured using the State Forest method and iFOVEA and Timbeter applications. The measurement included 23 broadleaf wood stacks (193.73 m^3^) and 14 coniferous wood stacks (149.23 m^3^).

### 2.2. Determination of Measurement Error of Wood Volume

**Figure 4:**
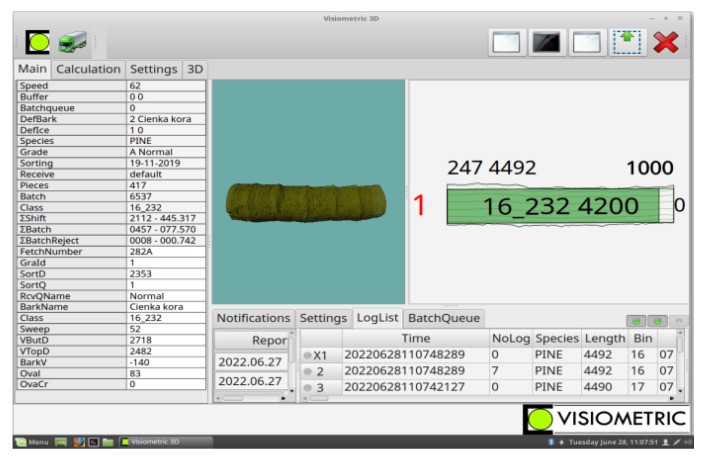
Example of wood measurement image from laser scanning on the log sorting line.

Verification of the accuracy of the determination of the volume of the large-sized wood load made by the forester and stated on the delivery note (m^3^) was made for randomly selected sawlog deliveries. The measurements were carried out at the sawmill using accurate laser scanning measurements during the pre-processing of the delivered wood on the log sorting line. The log sorting line was built in 2006 by Gosta Hedlund AB and the control system was supplied by RemaControl AB, now RemaSawco AB. The laser measurement system has been modified several times according to the requirements and guidelines of the sawmill. Each piece of round wood is scanned and measured. The image and results are displayed on a computer monitor at the line operator’s workstation (Figure 4).

Having the defined volumes of large-sized wood—Ls from laser scanning and medium-sized wood—S2 measured using iFOVEA and Timbeter applications, the secondary error percentage (Equation (4)) for these measurements was calculated in relation to a given reference volume (*V_r_*) calculated on the basis of manual measurement [49].

(4) $$\delta =\frac{\alpha }{V_{r}}\cdot 100\;\;\;$$

where

*δ*—absolute secondary error (%),

*α*—absolute error *α* = *V_iFOVEA/Timbeter/Mp_* − *V_r_* (m^3^),

*V_r_*—volume of wood determined by manual measurement (SF) (m^3^), and

*V_iFOVEA/Timbeter/Mp_*—volume determined by a given measurement method (iFOVEA, Timbeter and laser scanning—M_p_) (m^3^).

Due to the differences in the dendrometry parameters of broadleaf and coniferous wood [49] and the very different wood density conversion factors (Table 1) [44], the analyses were performed separately for broadleaf and coniferous wood piles.

### 2.3. Measurement of Characteristic Parameters of Wood Transports

The wood shipments were analyzed at the sites of three large wood consumers, processing different species and assortments. These were a furniture manufacturer’s sawmill purchasing Scots pine (*Pinus sylvestris* L.) large-sized wood (Ls) and two plants (pulp and paper and particleboard) purchasing medium-sized wood (S2). 

Wood volume in solid cubic meters (m^3^) delivered in the real transports analyzed was determined on the basis of delivery notes issued by foresters to the hauler. The weight of wood transported (Mg) was determined as the difference between the *GVW_actual_* of the transport set and the weight of the empty set (tare) (T), which was determined by weighing on stationary scales at the recipients’ sites (with an accuracy of 0.05 Mg) before and after unloading.

The field study analyzed 6300 (ca. 180 thous. M^3^) transports of Ls pine wood delivered to the sawmill and 1067 (ca. 32 thous. M^3^) transports of various S2 wood species (Table 1).

### 2.4. Comparison of the Real Total Gross Vehicle Weight of the Transport Set with That Established by Law

The volumes of transported round wood from delivery notes (m^3^), obtained from manual measurements (reference value), were converted by the average measurement error determined for a given photo-optical method (Me) for S2 (iFOVEA and Timbeter) and laser scanning on the log sorting line for Ls. All volumes of individual loads were then converted by the normative wood density coefficients (*Nc*) for the species analyzed (Table 2) according to the [44].

With a known weight of wood for a single delivery and the weight of an empty transport truck set (tare), the GVW was calculated according to Formulas (5) and (6).

The total gross vehicle weight of an transport set as determined by law (*GVW_law_*) is calculated by multiplying the load volume from delivery note (m^3^) by the normative factor (*Nc*) and adding the empty weight of the transport set (*T*).

(5) $$GVW_{law_{n}}=T_{n}+m_{n}^{3}\cdot Nc$$

where

*GVW_law_*—gross vehicle weight determined by law (Mg),

*T*—weight of empty transport set (tara) (Mg),

*m*^3^—wood volume obtained from manual measurements (m^3^),

*Nc*—normative wood density factor (Mg·m^−3^), and

*N*—data for the next transport.

Total gross vehicle weight of the transport set determined after taking into account measurement errors in *m*^3^ (*GVW_iFOVEA_, GVW_Timbeter_*) is calculated by multiplying the load volume from delivery note (*m*^3^) by the measurement error (*Me*), the normative factor (*Nc*) and the empty weight of the transport set (*T*):

(6) $$GVW_{iFOVEA_{n}/Timbeter_{n}/Mp_{n}}=T_{n}+m_{n}^{3}\cdot M_{e}\cdot N_{c}$$

where

$GVW_{iFOVEA_{n}/Timbeter_{n}/Mp_{n}}$*—*gross vehicle weight calculated after taking into account the measurement error of the wood volume in the respective iFOVEA, Timbeter and laser measurement method (*Mp*).

*Me*—mean secondary absolute error (Formula (4)) of measurement in the determination of wood volume for a given measurement method, for S2 wood loads separately for broadleaf and coniferous species (in hundredths)

Comparisons were made between all calculated GVW values and the real weights obtained from weighing the transport sets (*GVW_actual_*).

The obtained results were analyzed statistically with the use of the STATISTICA 12 package. The significance of differences was mainly determined using the Mann–Whitney test for two independent variables, as well as the Kruskal–Wallis test, and Dunn’s multi-sample rank mean comparison test (significant level was 0.05).

## 3. Results

### 3.1. Determining the Error in Measuring the Volume of the Stacks Medium-Sized Wood (S2)

**Figure 5:**
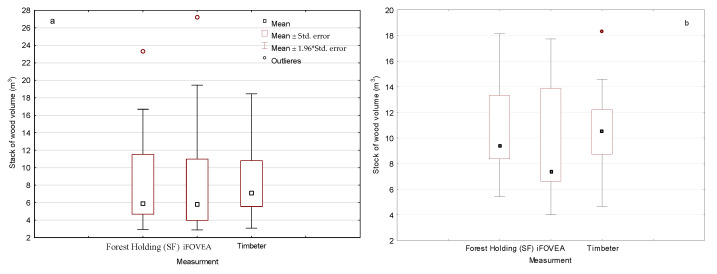
Statistical characteristics of the volume of a single pile by method of measurement. (**a**) broadleaf wood; (**b**) coniferous wood.

The statistical description characteristics of the volume of a single pile obtained by the tested methods are presented in Figure 5. The average volume of a broadleaf wood pile according to the measurement method in force in State Forest is 8.42 m^3^ (SD 5.24), and for the iFOVEA methods 8.53 m^3^ (SD 6.26) and Timbeter 8.27 m^3^ (SD 3.99). Such a large difference in results (Figure 5) affected the median values of 5.90, 5.80 and 7.09 m^3^ (Figure 5). Analysis with the Kruskal–Wallis test of the comparison of stack volumes by individual methods showed no statistically significant differences (*p* = 0.8401).

Coniferous stacks have a higher mean volume at 9.29–10.92 m^3^ and a median in the range of 7.35–10.51 m^3^ with a significantly smaller standard deviation of 3.28–4.32 m^3^ (Figure 5b). There were no statistically significant differences between the methods for determining stack volume (Kruskal Wallis test *p* = 0.0764).

**Figure 6:**
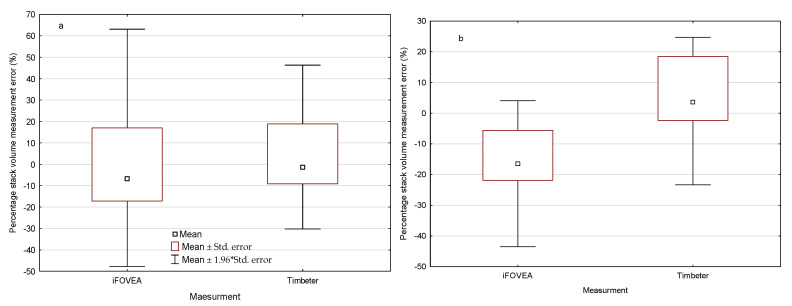
Statistical characteristics of single stack volume measurement errors by measurement method: (**a**) broadleaf wood; (**b**) coniferous wood.

For each pile, the measurement error of the pile volume using the iFVOEA and Timbeter methods was calculated in relation to the reference volume, and their characteristics are shown in Figure 6. The measurement error for broadleaf wood piles ranges from −47.67% to 63.16% (Figure 6a). There was no statistically significant difference in measurement error depending on the method used to determine the volume of broadleaf wood stacks (Mann–Whitney test with *p* = 0.291645). In comparison, the measurement error of the volume of coniferous wood stacks between methods differed significantly (*p* = 0.000304) (Figure 6b) with a spread of results from −43.31% to 24.72%.

Knowing the average volume of the S2 broadleaf and coniferous wood pile determined by a given method, the average percentage error of measurement for the iFOVEA and Timbeter methods was calculated in relation to the reference values. Determination of the volume of a broadleaf wood pile using the iFOVEA method had an average error of 1.34%, while the Timbeter method had an average error of −1.83%. In the coniferous wood pile measurement, the error is −12.82% and 2.41%, respectively. These error values were adopted for the simulation of the total gross vehicle weight of the transport set in Section 3.4.

### 3.2. Determining the Error in Measuring the Volume of the Large-Sized Wood (Ls)

**Figure 7:**
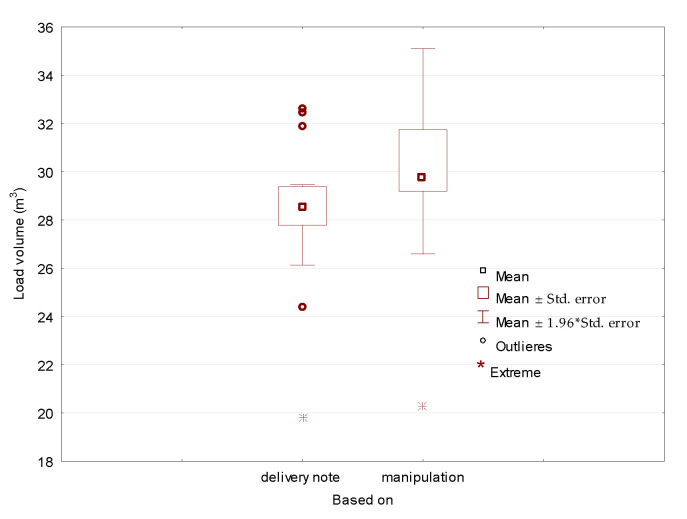
Characteristics of wood loads by delivery note and measurement using laser scanning.

Verification of the volume of load on the vehicle indicated on the delivery note (reference value) was carried out on the log sorting line (by laser scanning) for 20 transports. Detailed measurement showed larger loads by 0.10 m^3^ to 2.54 m^3^, giving a percentage error of 0.35% and 8.62%, respectively (Figure 7). The average measurement error is 6.10%. This value was used for further calculations. The Mann–Whitney test analysis confirmed the existence of statistically significant differences between the measurements (*p* = 0.0305).

### 3.3. Characteristics of Wood Loads and the Real Total Gross Vehicle Weight of the Transport Set (GVW)

The characteristics of Ls wood transports to the sawmill differ from S2 wood transports to both plants, with the greatest differences observed for GVW and weight of load with mean values for Ls of 50.68 and 30.55 Mg and for S2 of 46.42 and 26.87 Mg (Table 3). The Mann–Whitney test analysis confirmed statistically significant differences for all tested characteristics (*p* = 0.000) between large-sized and medium-sized wood transports.

**Figure 8:**
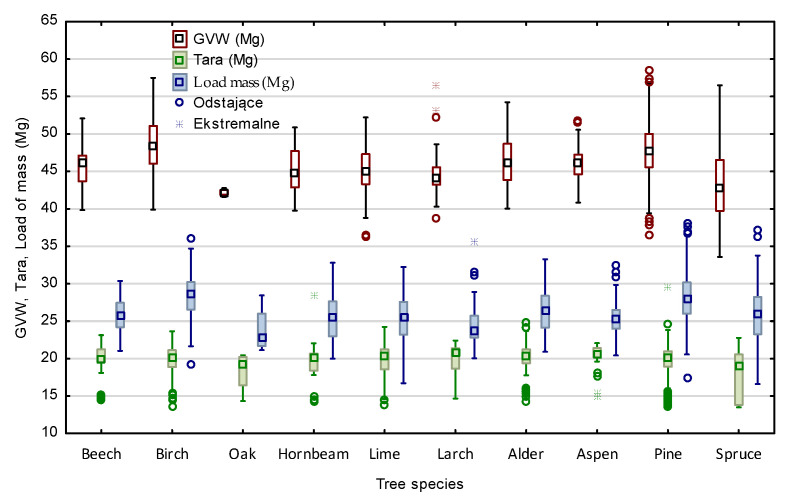
Characteristics of medium-sized wood transport parameters by wood species.

A detailed characteristic of S2 wood transports by species is presented in Figure 8. Analysis with the Kruskal–Wallis test (*p* = 0.000) of the tested parameters of the transports of each wood species showed statistically significant differences for all tested characteristics (Figure 8).

### 3.4. Comparison of Real GVW with Calculated Values for Medium-Sized (S2) Wood Transports

**Figure 9:**
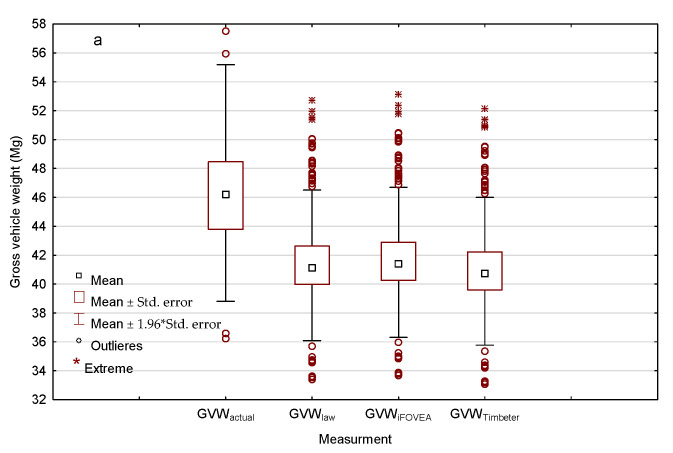

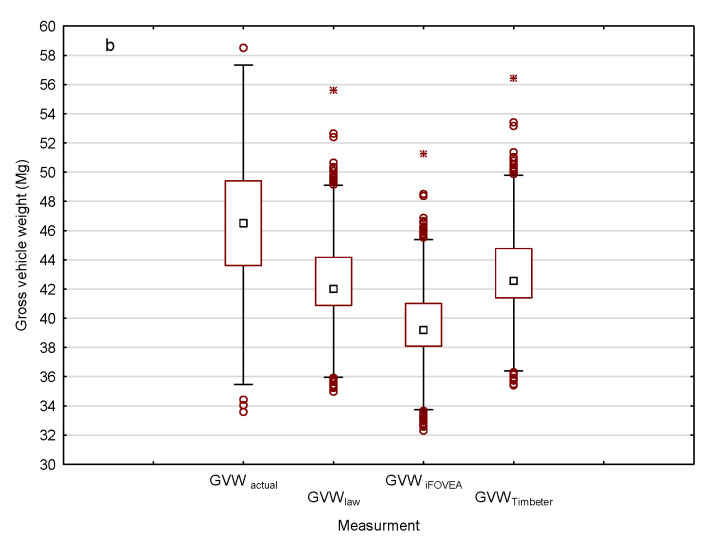
Comparison of the real total gross vehicle weight of the transport sets for S2 wood loads with the wood volumes of: (**a**) broadleaf wood and (**b**) coniferous wood, as determined by the legislation and the measurement method.

The real total gross vehicle weight of the transport sets (GVW_actual_) with S2 wood loads is significantly higher than the legislated weight (GVW_law_) and is different for broadleaf and coniferous wood (Figure 9). The average GVW_actual_ for broadleaf wood deliveries is 46.27 Mg (median 46.21) and coniferous is 46.52 Mg (median 46.50). The mean and median values of the calculated GVW (law, IFOVEA and Timberet) are similar, as confirmed by Dunn’s multi-sample rank mean comparison test (*p* = 0.451 and *p* = 0.112), which can be interpreted as no effect of the measurement method (iFOVEA, Timberet) on GVW_law_ (Figure 9a). The measurement error of the coniferous wood piles with the iFOVEA application (−12.82%) significantly underestimates the obtained GVW_law_ values (Figure 9b), and the results obtained are statistically significantly different (Kruskal–Wallis test *p* = 0.000).

**Figure 10:**
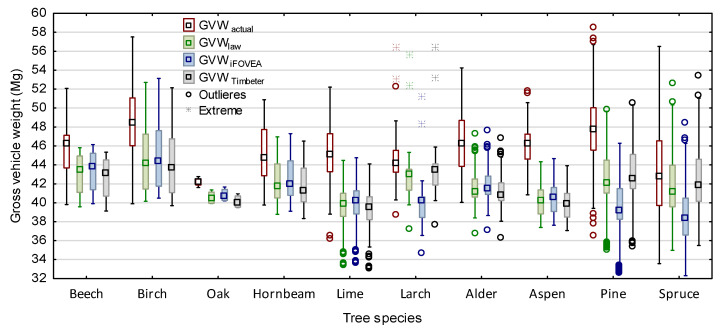
Comparison of the real total gross vehicle weight of transport sets for S2 wood loads with those established by law and the method of measuring wood volumes by species.

The different wood species have very different wood density conversion factors (0.67–0.98 Mg·m^−3^) [44]. As the calculated stack measurement errors depended on the tree species (Section 3.1), a corresponding analysis was also performed to illustrate the impact on GVW (Figure 10). For all species, the *GVW_actual_* transports differ from those obtained from the calculations. Particularly large differences are observed in the calculated GVW (law, iFOVEA and Timbeter) for wood loads of oak (*Quercus*), larch *(Larix*), pine (*Pinus*) and spruce (*Abies*).

### 3.5. Comparison of Real GVW with Calculated Values for Large-Sized (Ls) Wood Transports

**Figure 11:**
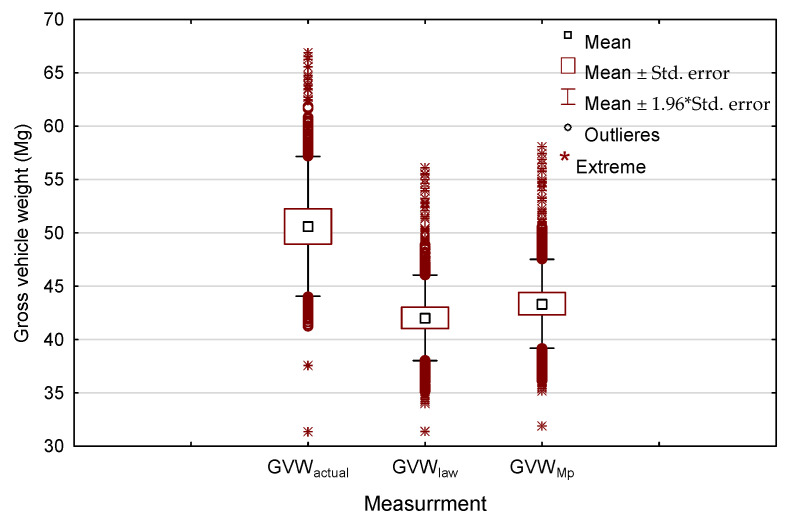
Comparison of the real total gross vehicle weight of transport sets for large-sized wood loads with that determined by legislation and accurate measurement by laser scanning.

The high real GVW_actual_ with a mean of 48.41 Mg (median 49.70) and a range of 31.50–66.70 Mg is the result of the conversion factor adopted for the weight per m^3^ of load in the legislation. Based on 6300 transports, with known wood volume and tare (empty weight of the transport set), after converting the load by 0.74 Mg·m^−3^ (conversion factor for pine, Table 2), the average GVW_law_ was 42.00 Mg, with a range of results from 31.39 to 56.09 Mg (Figure 11). The determined total gross vehicle weight of the transport set based on the conversion factor is on average underestimated by 20.7% and in extreme cases by as much as 40.3% compared to the real weight.

Taking into account that there is more wood on the truck than on the delivery note and a fixed average error of 6.10% in determining the volume of wood (Section 3.2), the average GVW_Mp_ is 43.33 Mg (range 31.88 to 58.03 Mg). This means that, even according to the legislation, the majority (95%) of the transport sets exceed the permissible total gross vehicle weight of the set defined at 40 Mg. The real total gross vehicle weights of the transport kit are still significantly higher by an average of 17.0%, which was confirmed by statistical analysis with the Kruskal–Wallis test showing statistically significant differences between the transport set weights (*p* = 0.000), and a multiple comparison of the mean ranks for all samples showed no pairs of similar results.

## 4. Discussion

Under Polish conditions, in accordance with the applicable standards, the permissible GVW is determined on the basis of the unloaded weight of the transport vehicle and the weight of the load, defined as the quantity of wood being transported in m^3^ adjusted by the appropriate table factor [3,25,32]. In this regard, the correct measurement of harvested wood is extremely important.

At present, the majority of timber measurements are taken manually. Increasingly, however, various technologies based on photo-optical techniques or laser scanning are being used to perform this activity. These use advanced calculation algorithms that enable measurements to be taken with high precision and efficiency [7,53,54]. The differences in measurement accuracy results between manual and photo-optical measurement, obtained during the study, are in most cases not statistically significant. This is also confirmed by the results obtained by other authors [7,10,15,16,17,18]. Similar relationships are also observed with laser scanning measurements. For example, the volume of the logs determined by the 2D laser scanning system was 0.4–0.5% higher than the volume determined by manual comparative measurement. The log volume determined by the 3D system was 2.5–5.5% lower than by careful manual measurement [9].

The main difference in stack wood measurements between the method currently used in the State Forests and the photo-optical method is the way in which the pile is analyzed. The measurement according to the technical conditions is based on the measurement of height, width and on conversion factors, the purpose of which is to convert spatial meters in bark into cubic meters of wood without bark [5]. These factors are applied identically across the country which can cause errors. As Bruchwald [49] notes, bark thickness is a characteristic that varies according to the part of the country and the age of the stand and the species of the [55,56]. Thus, it influences the result of the volume of the pile. Another variable is shape defects such as curvatures, flattening, convergence, girdle thickening or root inflows which, depending on their quantity in a given pile, may cause an increase in volume which the conversion factor does not take into account [57]. With the photo-optical method, each pile is analyzed individually. However, this method is not without its disadvantages. The stack must be properly prepared for measurement. In the case of an inappropriate location of the stack in the field, it is very difficult for the application to correctly identify the cross-cut foreheads of the logs at the very bottom of the stack [58].

In the accuracy of determining the volume of large-sized wood, it is important to correctly determine the diameter of the log without bark [49,55,56,59,60,61]. The authors [55,58,59] indicate that, depending on the method of measurement, there may be an underestimation of wood volume of 5–13%, which is consistent with the average error obtained in their study. When determining the volume of wood, it is also important to use the appropriate formula [49]. Its verification usually takes place by comparing the measurements with the results obtained by the method of water displacement technique (xylometer) [62,63,64].

**Figure 12:**
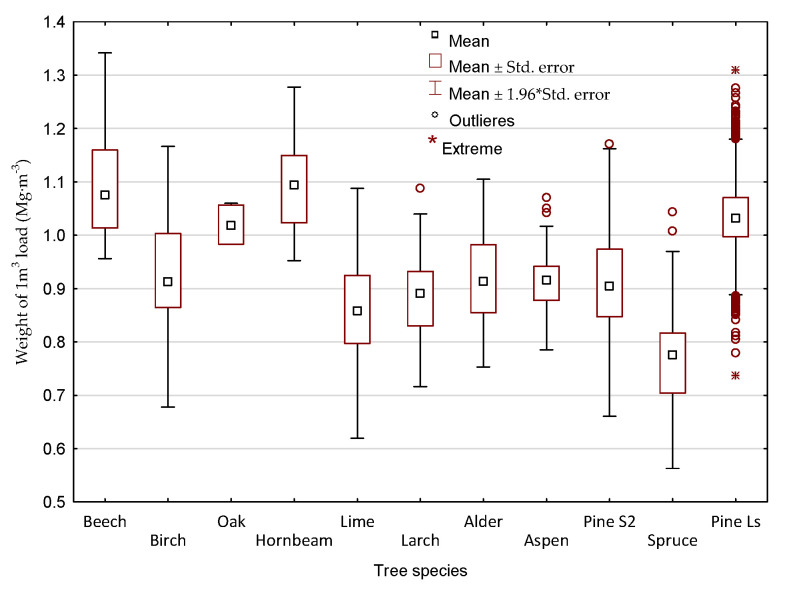
Statistical characteristic of weight of 1 m^3^ load by tree species. (Pine S2—for medium-sized pine loads; pine Ls—large-sized pine loads).

Differences between the real gross vehicle weight GVW_actual_ and the calculated (GVW_law_, GVW_iFOVEA_, GVW_Timbeter_) according to the wood density factor (Table 2) [44], both for coniferous and broadleaves are significant. The normative wood density coefficients does not take into account many factors that may have a significant effect on the density of wood, for example bark content, water content, different origin of the wood and season of the year [3,19,24,25,27,30,65]. The obtained mean values of weight of 1m^3^ load on the level 1.03 Mg·m^−3^ for large-sized (median 1.05) and 0.90 Mg·m^−3^ for medium-sized confirm the above statement. Analyzing the data for individual S2 wood species in detail, it can be seen that for some of the results for birch (*Betula pendula)*, lime (*Tilia*), larch (*Larix*), pine (*Pinus*), spurce (*Abies*), values similar to or lower than the tabulated values were obtained (Table 2) (mean ± 1.96*st. error—first quartile) (Figure 12). However, for large-sized pine timber loads, these values are usually higher, as also pointed out by other authors [21,28,29,30].

The obtained average GVW_law_ values of 42.00 Mg for large-sized transports (Figure 11) and 41.69 Mg and 42.02 Mg for S2 broadleaf and coniferous wood (Figure 9) indicate that, according to Polish legislation, vehicles are overloaded at the loading stage (handing over of wood by the forester). Only approximately 12% of large-sized and 20% of S2 timber transports had a GVW_law_ ≤ 40 Mg. This may be related to the highly variable empty weight of the transport set (tare) 13.50–29.52 Mg (Table 3), as well as the possibility of different combinations of truck or truck-trailer combination [3,20,22,24,25,66]. This phenomenon has also already been noted in previous studies [67].

## 5. Conclusions

Where the gross vehicle weight for wood transport is standardized and calculated based on conversion factors, include the volume of the round wood load, the determination of its correct measurement is extremely important.

Currently, there are a number of computer programs and applications for mobile phones on the market, based on advanced calculation algorithms, which enable this process to be carried out much more quickly than manual measurement.

This study showed that the results of pile volume measurements of medium-sized wood using iFovea and Timbeter applications are not statistically significantly different from traditional (manual) measurements. The results of measuring the volume of wood for large-sized wood from laser scanning are statistically significantly different from the manual measurement.

The real total gross vehicle weights of transport sets under Polish conditions are often much higher than those established by law. This is because the normative wood density coefficients do not take into account the bark content and seasonal variations in wood moisture content. Therefore, it is reasonable, to take action to adjust these factors to make them more reflective of real values.

## Figures and Tables

**Table 1:** Volume and amount of S2 wood deliveries by tree species.

Tree Species	Amount of Deliveries	Delivered Quantity m^3^
Birch (*Betula pendula*)	85	2619.73
Beech (*Fagus sylvatica*)	33	780.42
Oak (*Quercus*) *	4	93.50
Hornbeam (*Carpinus betulus*)	25	584.45
Lime (*Tilia*)	127	3747.24
Larch (*Larix*)	72	1975.46
Alder (*Alnus*)	144	4135.40
Aspen (*Aspen*)	44	1229.57
Pine (*Pinus*)	414	12,948.63
Spruce (*Picea*)	119	4039.31
Total	1067	32,153.71

* Excluded from statistical analyses (sample too small).

**Table 2:** Normative wood densities of selected species.

Lp.	Tree Species	Wood Density kg·m^−3^
1	Birch (*Betula pendula*)	810
2	Beech (*Fagus sylvatica*)	980
3	Oak (*Quercus*)	950
4	Hornbeam (*Carpinus betulus*)	960
5	Lime (*Tilia*)	670
6	Larch (*Larix*)	830
7	Alder (*Alnus*)	750
8	Aspen (*Aspen*)	710
9	Pine (*Pinus*)	740
10	Spruce (*Abies*)	720

Source: [Regulation …2012] [44].

**Table 3:** Characteristic of wood deliveries parameters.

Measure	Assortment	Mean	SD	Min	Max	Q1	Median	Q3
*GVW_actual_* (Mg)	Ls S2	50.68 46.42	2.74 3.94	31.35 33.58	66.90 58.50	48.95 43.74	50.60 46.36	52.25 49.04
Load volume (m^3^)	Ls S2	29.55 30.13	2.21 4.25	11.00 18.56	43.50 44.88	28.36 28.00	29.26 28.78	30.47 33.12
Weight of load (Mg)	Ls S2	30.55 26.87	2.67 3.45	8.10 16.60	45.92 38.10	28.90 24.48	30.30 26.72	32.00 29.08
Weight of empty set (tara) (Mg)	Ls S2	20.13 19.55	1.66 2.39	13.50 13.48	24.15 29.52	19.70 18.76	20.40 20.20	21.00 21.12
Weight of 1 m^3^ load (Mg·m^−3^)	Ls S2	1.03 0.90	0.06 0.11	0.74 0.50	1.31 1.34	1.00 0.83	1.05 0.90	1.07 0.97

Notes: SD—standard deviation; Q1—first quartile; Q3—third quartile.
